# Developing feasible healthy diets for Ethiopian women of reproductive age: a linear goal programming approach

**DOI:** 10.1017/S1368980023001374

**Published:** 2023-10

**Authors:** Tesfaye Hailu Bekele, Maike van Rooijen, Johanna C Gerdessen, Inge D Brouwer, Edith JM Feskens, Laura Trijsburg, Dawit Alemayehu, Jeanne HM de Vries

**Affiliations:** 1 Ethiopian Public Health Institute, Addis Ababa, Ethiopia; 2 Division of Human Nutrition and Health, Wageningen University and Research, Wageningen, The Netherlands; 3 Group Operations Research and Logistics, Wageningen University and Research, Wageningen, The Netherlands

**Keywords:** Diet modelling, Optimisation, Continuous fasting, Intermittent fasting, Cost of diet

## Abstract

**Objective:**

To develop a healthy diet for Ethiopian women closely resembling their current diet and taking fasting periods into account while tracking the cost difference.

**Design:**

Linear goal programming models were built for three scenarios (non-fasting, continuous fasting and intermittent fasting). Each model minimised a function of deviations from nutrient reference values for eleven nutrients (protein, Ca, Fe, Zn, folate, and the vitamins A, B_1_, B_2_, B_3_, B_6_, and B_12_). The energy intake in optimised diets could only deviate 5 % from the current diet.

**Settings:**

Five regions are included in the urban and rural areas of Ethiopia.

**Participants:**

Two non-consecutive 24-h dietary recalls (24HDR) were collected from 494 Ethiopian women of reproductive age from November to December 2019.

**Results:**

Women’s mean energy intake was well above 2000 kcal across all socio-demographic subgroups. Compared to the current diet, the estimated intake of several food groups was considerably higher in the optimised modelled diets, that is, milk and dairy foods (396 *v*. 30 g/d), nuts and seeds (20 *v*. 1 g/d) and fruits (200 *v*. 7 g/d). Except for Ca and vitamin B_12_ intake in the continuous fasting diet, the proposed diets provide an adequate intake of the targeted micronutrients. The proposed diets had a maximum cost of 120 Ethiopian birrs ($3·5) per d, twice the current diet’s cost.

**Conclusion:**

The modelled diets may be feasible for women of reproductive age as they are close to their current diets and fulfil their energy and nutrient demands. However, the costs may be a barrier to implementation.

Ethiopia has prioritised improving the diet to combat malnutrition and non-communicable diseases (NCD)^(1,2)^, and special focus should be given to the diet of women, future mothers and young children^(3,4)^. The nutritional status of Ethiopian women is currently characterised by inadequate intake of micronutrients, attributable to a lack of nutrition knowledge on diet quality, low access to a healthy diet and heavy daily workload^(5,6)^. Dietary recommendations for Ethiopian women of reproductive age are needed to initiate the dietary transition towards a healthier diet and to meet their nutrient requirements for the prevention of malnutrition and diet-related NCD. However, dietary recommendations can only be feasible if the food price and affordability of a healthy diet are taken into consideration^(7)^.

Differences in women’s nutritional status and dietary intake between urban and rural areas and between the fasting and non-fasting seasons have been well recognised in Ethiopia^(8,9)^. Between 1990 and early 2010, the prevalence of being underweight had reduced more in urban areas than in rural areas^(10)^. Moreover, the prevalence of overweight and obesity was consistently higher in urban areas than in rural areas^(11)^. Therefore, differences in nutritional status and dietary intake between areas should be considered while developing dietary recommendations. In addition, women’s health is also influenced by fasting^(12)^, especially in the Ethiopian Orthodox Church, which is attended by 43 % of the total population. In Ethiopia, fasting refers to avoiding animal foods (such as meat, dairy products and eggs) and not eating or drinking before 15.00. There are generally two types of fasting: continuous fasting (fasting for 2–8 weeks) and intermittent fasting (2 d of fasting per week on Wednesdays and Fridays). The annual number of fasting days can amount to up to 140 and range from only single days to a more extended period of consecutive days^(13)^. Because of its high frequency, fasting periods should be considered when planning Ethiopian diets^(14)^.

Developing the Ethiopian Food-Based Dietary Guidelines (FBDG) is an important part of the public health strategy. FBDG recommends the appropriate amount of foods and food groups that need to be consumed daily by certain subpopulations with specific nutrient requirements^(15)^. Mathematical dietary optimisation models support the development of such guidelines by creating food plans that most closely resemble current dietary habits while meeting predetermined nutritional, cost and sometimes environmental constraints^(16)^. Previously, expert consultations were used to develop optimal diets using a ‘trial-and-error’ approach, but nowadays, more rigorous mathematical modelling allows this process to be done more efficiently^(17)^.

Both linear and non-linear programming models have been used to inform dietary recommendations^(18)^. In Asia, for example, Anderson and Earle (1983) used goal programming to balance nutrients in the daily diet of the Thai people^(19)^. In North America, Foytik (1981) used linear programming to establish a new diet plan for American low-income families^(20)^. Since then, modelling has been used more often among different population groups^(21)^. In the current study, a linear goal programming technique was used, derived from the model developed by Gerdessen and de Vries^(22)^. The goal programming technique was used in previous studies to optimise individual diets in the Netherlands and Tanzania^(23,24)^. This study aims to develop a healthy diet for Ethiopian women of reproductive age that closely resembles their current eating habits while meeting predetermined nutrients and cost constraints using mathematical modelling.

## Methods

Tailored linear programming models for non-fasting, continuous and intermittent fasting scenarios were created. All models were solved with FICO Xpress 8.10. This section explains the models textually, while the mathematical formulation is in Appendix 1.

### Study population

The models were generated using data obtained from 24-h dietary recall (24HDR) information collected over two non-consecutive days. The 24HDR data were gathered between November and December 2019 from 494 women of reproductive age who were not pregnant or lactating and resided in five Ethiopian regions (Amhara, Oromia, Southern Nation and Nationalities, Tigray, and Addis Ababa). In these five regions, 100 households per region were selected. Of these 100 households, fifty households were from low agriculture productivity district areas (based on the agriculture office annual report) and fifty households from high agriculture productivity district areas. In Addis Ababa (the capital city), fifty households were chosen from urban slum areas and fifty were assigned to areas with better living conditions. The households were selected using systematic random sampling from the selected district’s household lists. One woman of reproductive age from each selected household participated in the nutrition study. If there are more than one women who qualified for the inclusion criteria, the senior woman is included in this study.

### Data collection, market survey and additional data input

A daily schedule was used to assign 24HDR to different days of the week so that intake data of all days of the week were equally presented at the population level. A multiple-pass 24HDR method was used to improve recall^(25)^. The 24HDR questionnaire and prompt techniques were applied for the in-person interview. Data collectors (bachelor’s and master’s graduates in nutrition or public health), trained in conducting interviewer-administered 24HDR, collected a quick list of all foods consumed in the previous 24 h from study participants. There was no continuous fasting season during the data collection period.

In the 24HDR, a list of consumed foods was recorded quickly, and after that detailed information about each dish, including its recipe and types of foods used, was collected. To improve the accuracy of ingredient measurement, all ingredients were weighed using a digital food scale after measuring a known weight. The measurements were checked for accuracy by measuring each food item twice. If an ingredient was unavailable or impossible to measure, substitutes such as water were weighed and converted using appropriate conversion factors^(25)^. Standard portion sizes were used when actual food measurement was not feasible or appropriate. The data were thoroughly cleaned to address missing values, conversion factors for liquid foods measurements and raw-to-cooked conversions. Information on the conversion and edible factors of the foods was gathered during the 24-HDR data collection, while raw-to-cooked factors were taken from Ethiopia’s 2011 National Food Consumption Survey. The data were then linked to Ethiopia’s food composition table, which was revised during the 2011 National Food Consumption Survey^(26)^. Finally, the food intake was converted into energy and nutrients using Compleat software (www.compleat.nl). Additionally, socio-demographic and household food security^(27)^ information was gathered. The data collectors collected price information for all ingredients consumed by the women from three different retailers in the community’s nearby main marketplace in the study area. When the retailer used the local unit of measurement for selling, the food items were weighed using a food weighing scale, but when they used standard units of measurement such as kilograms or litres, the price was verbally recorded instead. Two community markets were visited per region. We averaged the prices of the markets and converted them to a price per 100 grams of food per region.

The food items from the 24HDR were divided into twelve categories based on the women’s minimum diet diversity (MMD-W) score and adapted to the actual consumption, as detailed in Appendix 2. The MDD-W is a validated tool most often used to assess diet quality and food grouping to report dietary intake^(28)^. The average nutrient reference values for the three age categories (15–17, 18–24 and 25–50 years of age) were derived from harmonised reference values proposed by Allen et al.^(29)^, and these values were used to derive the nutrient reference values in consultation with the Ethiopian FBDG committee. The specific nutrient reference values used in the model are described in Table 1.

**Fig. 1:**
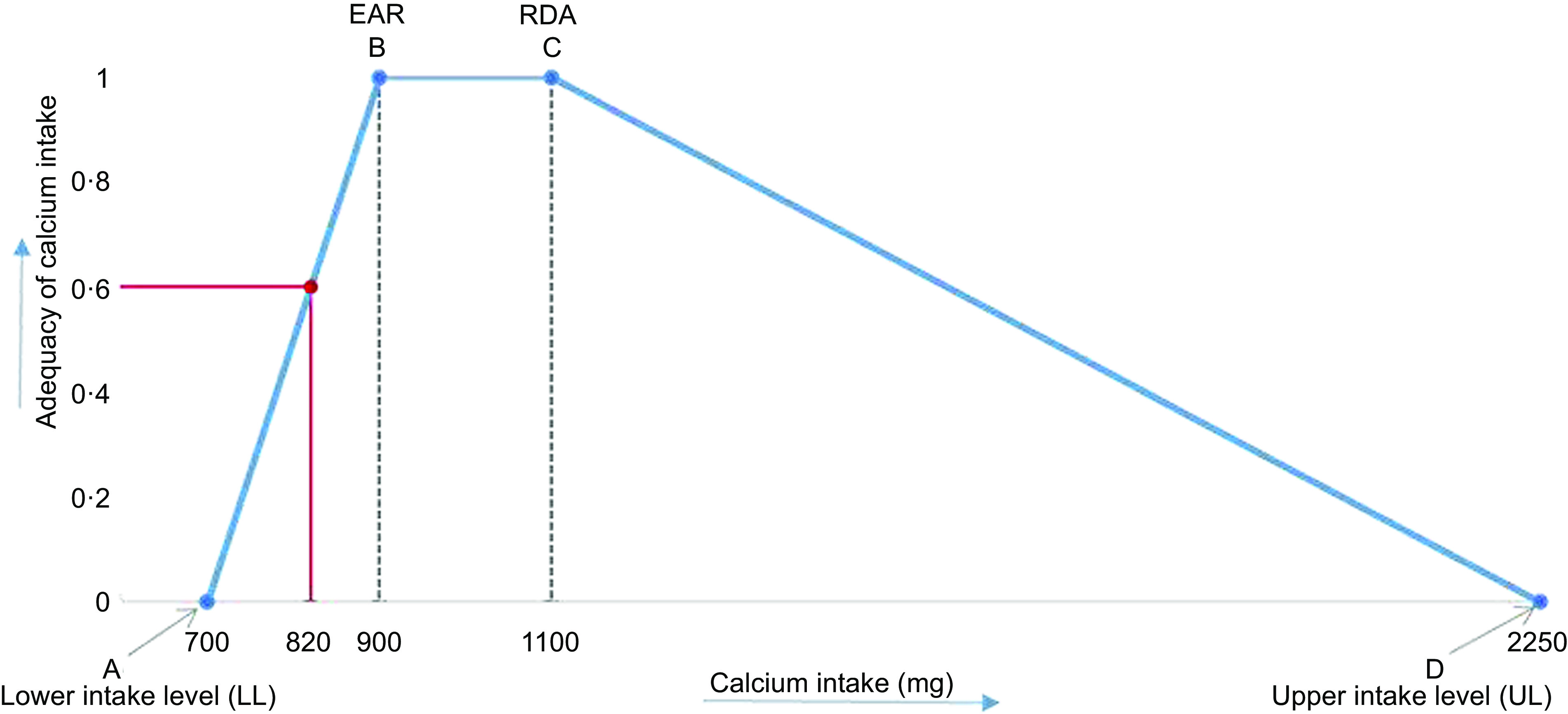
Adequacy curve for calcium intake (for calcium, EAR = 900 mg/d and RDA = 1100 mg/d). EAR, estimated average requirement

**Table 1:** Nutrient reference values considered in the diet modelling for women of reproductive age (15–49 years)

	Nutrient	EAR	RDA	UL
1.	Protein (g/kg/d)	0·68	0·82	2·00
2.	Ca (mg/d)	900	1100	2250
3.	Fe (mg/d)	8·03	17·0	45·0
4.	Zn (mg/d)	6·97	8·30	25·0
5.	Vitamin A (µg RAE/d)	495	700	3000
6.	Thiamin (mg/d)	0·90	1·07	–
7.	Riboflavin (mg/d)	0·90	1·07	–
8.	Niacin (mg/d)	11·0	14·0	35·0
9.	Vitamin B_6_ (mg/d)	1·07	1·27	25·0
10.	Folate (µg/d)	323	400	1000
11.	Vitamin B_12_ (µg/d)	2·0	2·4	–

EAR, estimated average requirement; RAE, retinol activity equivalents, RDA, recommended daily allowance, UL, upper intake level.

Based on Allen et al., 2020^(29)^. Explanation of nutrient reference values: EAR (intake at which an individual’s risk of inadequacy is 50 %); RDA (intake at which an individual’s risk of inadequacy is 2–3 %); tolerable UL (the intake at which adverse effects may occur).

### General description of the linear goal programming model

The current diets of the Ethiopian women in this study’s sample were optimised by running the diet model developed specifically for this study on each woman. The diet model was formulated according to the linear goal programming approach described in Appendix A of Gerdessen & De Vries^(22)^. This approach aims to find a so-called Pareto-optimal solution, in which the adequacy of one nutrient’s intake cannot be improved without compromising the adequacy of another nutrient’s intake. The approach finds a Pareto-optimal diet for each woman by quantifying the extent to which the diet violates the nutrient intake constraints and minimising a so-called achievement function of these violations. It is assumed that the adequacy of a diet is determined by the adequacy of the bottleneck nutrient, that is, the nutrient that has the most inadequate intake. Therefore, in this research, the achievement function minimises the largest unwanted nutrient intake deviation (MinMax) (see Appendix 1). To ensure optimised diets closely resemble observed diets, upper bounds are defined for the percentage differences in food intake in grams per food group and food subgroup between the observed diet and optimised diet.

### Quantifying deviations from nutrient reference values

To quantify the deviations from nutrient reference values, constraints are formulated via adequacy curves^(22)^. As an example, the adequacy curve of Ca is shown in Fig. 1. The four characteristic points are defined as follows: A is the lower intake level (LL) below which an intake could lead to health risks in most individuals; B is the estimated average requirement (EAR) that meets the nutrient needs of half of the healthy individuals; C is the recommended daily amount which is sufficient for nearly all people and D is the upper intake level (UL) that is unlikely to pose a risk of adverse health effects. Nutrient intakes below the LL or the UL are fully inadequate (adequacy equals 0), and nutrient intakes between EAR and RDA are fully adequate (adequacy equals 1). However, as most current diets are too poor to optimise to full adequacy, we try to increase the nutrient intakes so that they get closer to the level of the EAR. Between the LL and the EAR, a linear increase of the adequacy is assumed, and between the RDA and the UL a linear decrease is assumed for all selected micronutrients. The Ca intake in Fig. 1 is at 60 % between the LL and EAR.

### Constraints to limit the deviation of food consumption from the current diet

To keep the optimised diets attainable, constraints are set to limit the deviation of food consumption from the current diet in terms of food groups and food subgroups. If the current diet is not adequate, a low allowed deviation leads to realistic outcomes but lower dietary adequacy. A high allowed deviation leads to non-realistic outcomes but higher dietary adequacy.

On the food group level, the total amount of foods consumed in the food group in the optimised diet was allowed to deviate 10 % from the current consumption. On the food subgroup level, this amount was allowed to deviate by up to 15 %. For example, if a woman consumed 40 g of food subgroup *Other fruit* in her current diet, the consumption of *Other fruit* in her optimised diet could vary from 34 g to 46 g. As long as this constraint is met, all of the food items that belong to this food subgroup are interchangeable. A more extensive numerical example is shown in Appendix 3. According to a sensitivity analysis, allowing more deviation than described above did not improve the adequacy of the diet enough to justify an increased deviation from the current diet. Moreover, a constraint was set on the energy intake. As this study did not aim to change women’s energy intake, the energy intake in optimised diets could only deviate 5 % from the current diet.

### Handling barely consumed food groups

The consumption data showed that food groups such as nuts and seeds, milk and dairy products, fish and shellfish, meat and eggs, and fruits were barely consumed by the sample population. For these food groups, the constraints that limit the differences between current and optimised diet would prevent that these foods are included in the optimised diet. To assess the impact on micronutrient intakes of including these food groups in optimised diets model runs were set up that allowed the inclusion of these food groups, not by using constraints but by defining lower and upper bounds for each of them. These bounds were based on the Global Burden of Disease (GBD) study recommendations^(30)^, and as shown in Table 2, models included lower bounds of 0 %, 50 %, or 75 % and upper bounds of 50 %, 75 %, or 100 % of the total grams/d of the GBD recommendations, respectively. This was done to show what would happen to the adequacy of diets if food items from uneaten food groups are included in the diets. There were no additional constraints on the division of these recommended grams on subgroup level. Furthermore, a model run was set up in which no GBD recommendations were applied.

**Table 2:** Assumptions used for barely consumed foods groups, based on the Global Burden of Disease (GBD) daily food group consumption recommendations^(30)^ included in each of the model runs (0–50 % GBD, 50–75 % GBD and 75–100 % GBD)

Food group name*	GBD (g/d)	0–50 % GBD		50–75 % GBD		75–100 % GBD	
		Lower bound (g/d)	Upper bound (g/d)	Lower bound (g/d)	Upper bound (g/d)	Lower bound (g/d)	Upper bound (g/d)
Nuts and seeds	21	0	10	10	15	15	21
Milk and dairy products	435	0	218	218	326	326	435
Fish and shellfish **+** meat and eggs†	23	0	11	11	17	17	23
Fruits	250	0	125	125	188	188	250

* For the food groups not mentioned here, the number of grams included in the optimised diet is allowed to deviate 10 % from the number of grams in the current diet.

† The GBD recommendations of the food groups ‘fish and shellfish’ and ‘meat and eggs’ are combined.

### Fasting models

Models were set up to account for non-fasting, continuous fasting and intermittent fasting periods. The non-fasting model is the diet model as explained so far. For the continuous fasting model, all 24HDR data were used as input, but animal-based foods were excluded from optimised diets, since the majority of the study population consumed a plant-based diet during fasting. All 24HDR data were used as input, because in practice the study population barely consumed any animal products on non-fasting days either. The energy requirements remained unchanged. Furthermore, vitamin B_12_ and Ca constraints were disregarded, as it turned out to be impossible to meet their requirements without consuming animal-based foods.

The intermittent fasting model composed a weekly diet including two fasting days (Wednesday and Friday)^(13)^ and five non-fasting days (Monday, Tuesday, Thursday, Saturday and Sunday). Animal-based foods were excluded from diets on fasting days. The main difference with the other two models is that Ca and vitamin B_12_ requirements were considered on a weekly and not on a daily basis. In that way, inadequate Ca and vitamin B_12_ intakes on fasting days could be compensated on non-fasting days.

### Analysing the impact of the model on intake and cost

Additional statistical analysis of the optimised diet results was undertaken using Stata 17. The mean or median consumption and the prevalence of inadequate nutrient consumption were compared for current and modelled diets. Mean energy intake differences across socio-demographic subgroups were analysed using a one-way ANOVA. Energy and nutrient intake differences between the current and modelled diets were analysed using Dunn’s test.

## Results

The two 24HDR were collected during the harvest season, when food is abundant, with the intention to capture the different foods available in Ethiopia. This resulted in an overall high energy intake (above 2000 kcal) (Table 3). The youngest women, between the ages of 15 and 25 years, had a higher energy intake than the older age group, and energy intake was higher in households with a higher level of food security. Religion was also associated with total energy intake, possibly due to more frequent holidays during the data collection period than other months of the year.

**Table 3:** Mean (sd) and median energy (interquartile (IQR) range) intake in the current diet of women of reproductive age by socio-demographics based on a 2-d 24HDR

*n* 494	*n*	Energy (kcal)				
		Mean	sd	Median	IQR	*P*-value*
Age group (years)						
15–25	126	2857	985	2703	1385	<0·001
26–35	200	2743	1009	2602	1401	
36–49	162	2448	882	2341	1206	
Women’s level of education						
No education	253	2732	979	2632	1312	
Primary	153	2690	956	2569	1338	0·169
Secondary	68	2499	1048	2428	1167	
College and above	14	2315	573	2370	573	
HH family size						
≤3	87	2781	990	2597	1233	0·205
4–5	155	2567	959	2480	1463	
≥6	246	2705	977	2598	1265	
HH food insecurity						
Secure	131	2801	972	2632	1316	0·009
Mild insecure	133	2820	955	2692	1071	
Moderate insecure	116	2542	979	2481	957	
Sever insecure	108	2484	960	2278	2127	
Religion						
Orthodox	350	2786	986	2632	1247	<0·001
Muslim	58	2437	963	2030	1186	
Protestant	69	2222	725	2367	1191	
Other	11	3003	1167	2958	1547	

* *P*-values are based on one-way ANOVA to determine the mean difference between categories within groups.

### Comparison of the current diet with modelled diets

A total of 494 women had complete data from two 24HDR. In addition, for 461 women in Model 1, 415 women in Model 2 and 425 women in Model 3, a feasible diet was achieved for the target nutrients as defined in Fig. 1. Table 4 shows the median nutrient intake and percentage of women with an inadequate intake of nutrients in the current diet and the three modelled diets. For riboflavin, vitamin B_6_ and Fe, < 10 % of women had an inadequate current dietary intake. The median values for the other nutrients, such as vitamin A, vitamin C, niacin and folate, showed an improvement in the three modelled diets compared to the current diet. Because Ca and vitamin B_12_ were not constrained in Model 2 (continuous fasting), the percentage of people below EAR of these nutrients was comparable with the current diet. Similarly, for Zn, only a slight increment in the percentage of women with inadequate intakes was seen for Model 2 compared to the current diet.

**Table 4:** Daily median (interquartile (IQR) range) intake of nutrients and percentage of women with inadequate intakes for current and optimised diets

Micronutrients in mg	Current diet			Model 1 or non-fasting diet (*n* 461)			Model 2 or continuous fasting diet (*n* 415)			Model 3 or intermittent fasting diet (*n* 425)¶			EAR|| (mg/d)
	Median	IQR	Percent (<EAR)	Median	IQR	Percent (<EAR)	Median in mg	IQR in mg	Percent (<EAR)	Median	IQR	Percent (<EAR)	
Thiamine (vit B_1_)	1·42	0·79	22·9	1·30	0·43**	12·6	1·39	0·56	10·7	1·41	0·48	7·1	1·0
Riboflavin (vit B_2_)	1·36	0·91	8·90	1·98	0·54**	0·0	1·40	0·59*	0·0	1·95	0·58**	0·0	0·7
Niacin (vit B_3_)	12·8	8·12	14·2	14·8	6·35**	0·0	18·9	8·22**	0·0	16·5	6·36**	0·0	8·0
Vitamin B_6_ †	2·14	1·10	5·30	1·89	0·76**	0·0	1·93	0·76**	0·0	2·10	0·92	0·2	0·9†
Vitamin B_12_ †	0·00	0·18	94·7	2·42	0·51**	0·0	0·11	0·07**	84·0	2·00	0·00**	5·1	1·6†
Vitamin A†,‡	95·6	649·3	62·8	495	0·00**	0·0	495	0·00**	0·0	495	0·00**	0·0	290†,‡
Vitamin C	30·8	28·0	74·1	122	33·5**	0·0	109	36·3**	0·0	104	38·6**	0·0	45·7
Ca	591	414·1	79·6	900	55·4**	6·9	700	0·00	77·5	900	0·00**	12·8	900
Fe	23·3	13·5	5·70	19·6	6·0**	0·6	20·3	5·40**	0·0	23·1	4·30	0·2	8·0
Folate†	418	308·8	32·4	323	26·9**	7·5	323	13·8**	12·4	347	76·3*	8·5	323†
Zn	13·2	8·63	16·2	8·60	2·83**	8·7	7·70	1·89**	19·8	9·61	3·96**	5·3	7·0

*P < 0·05 and **P < 0·001 indicate mean difference between the current and modelled diet from unadjusted and adjusted Dunn’s test (using Holm–Sidák method).

† μg.

‡ Retinol activity equivalence.

|| Allen et al., 2020.

¶ Model 3 or intermittent fasting diet – an average of five non-fasting and two fasting days per week.

**Fig. 2:**
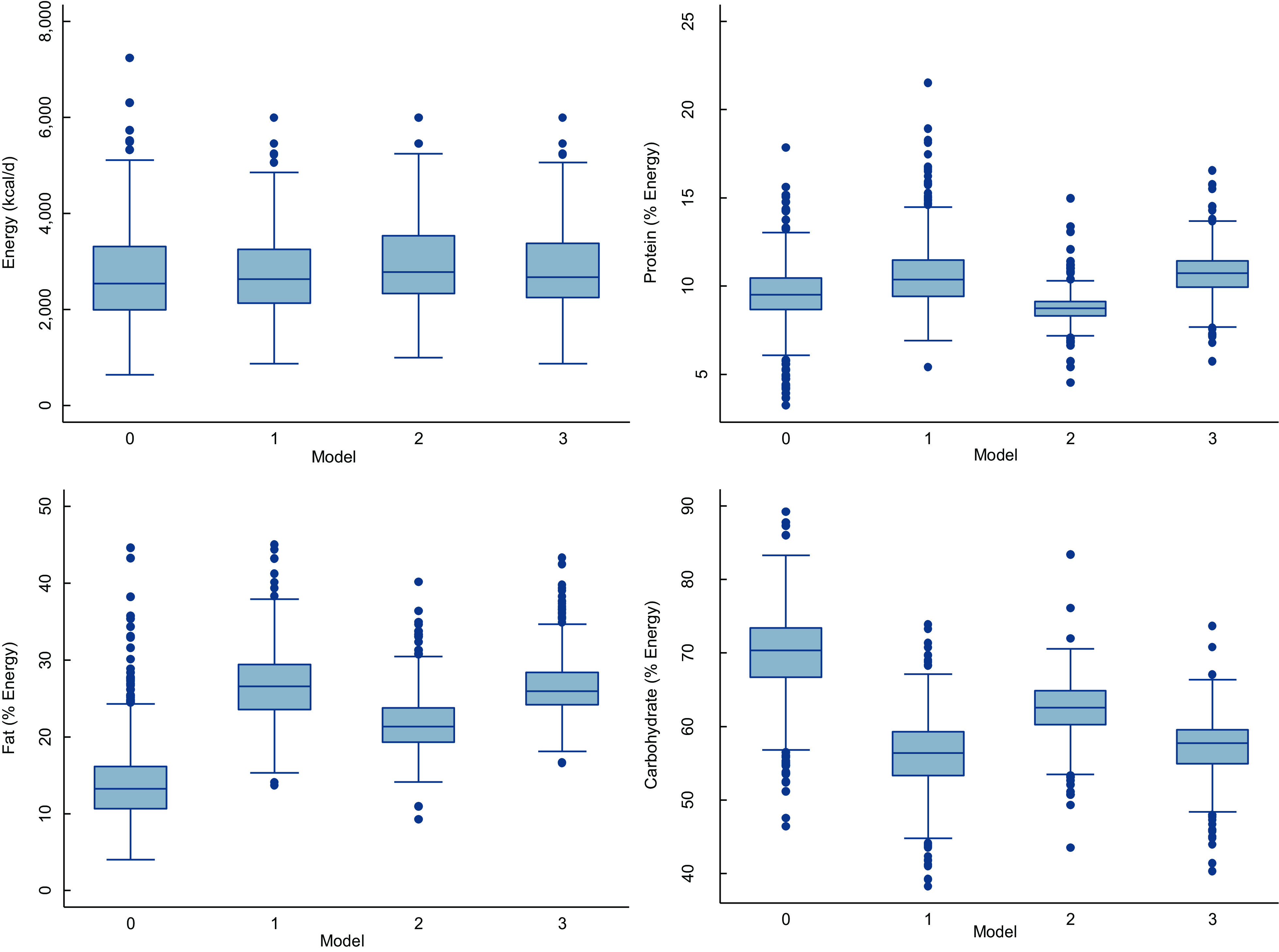
The distribution of energy and macronutrients in the current and modelled diets (X-axis represents the different models and Model 0, which is the current diet)

Figure 2 shows the current and modelled diet’s median energy and macronutrient intake. By design, the current and modelled diets’ median daily energy intake did not differ. In terms of daily protein consumption, Models 1 and 3 (non-fasting and intermittent fasting) but not Model 2 (continuous fasting) resulted in higher intakes than the current diet. The current diet had the lowest median fat intake; less than 1 % of the population had a fat intake of 40–45 energy per cent, while 89 % had a fat intake below 20 energy per cent. In the modelled diets, the majority of the study population consumed less than 20 % of their total energy fat (86 % for Model 1, 55 % for Model 2 and 83 % for Model 3). The median daily carbohydrate intake of the modelled diets was lower than that of the current diet, especially for Models 1 and 3.

Table 5 shows the results per food group. The contribution of nuts and seeds was higher in the modelled diets than in the current diet. The recommended amount of milk and dairy products in Model 1 (396 g/d) and Model 3 (298 g/d) was much higher than in the current diet (31 g/d). In contrast, the recommended amount of vegetables in Model 1 (129 g/d), Model 2 (135 g/d) and Model 3 (132 g/d) did not deviate much from the current diet (137 g/d). The contribution of fruits increased in all modelled diets compared to the current diet.

**Table 5:** The proposed amount in grams/d in Ethiopia’s modelled diets for women of reproductive age and their current intake at the food groups level

Food groups	Current diet	The recommended amount obtained from modelled diet		
		Model 1 or non-fasting diet	Model 2 or continuous fasting	Model 3 or intermittent fasting
1. Grain, white roots and tubers	608	568	611	588
2. Pulses	91	88	92	90
3. Nuts and seeds^*^	1	20	20	21
4. Milk and dairy foods^*^	31	396	0	298
5. Meat, fish and egg^*^	12	22	0	16
6. Fruits^*^	7	195	202	194
7. Vegetables	137	129	135	132
8. Fats and oils	15	17	15	16
9. Sugar-sweetened beverages (SSB)	30	29	30	28
10. Beverages, spices and other	346	322	338	337

* The recommended amounts that require a big shift from the current diet.

### The cost of a healthier diet

**Fig. 3:**
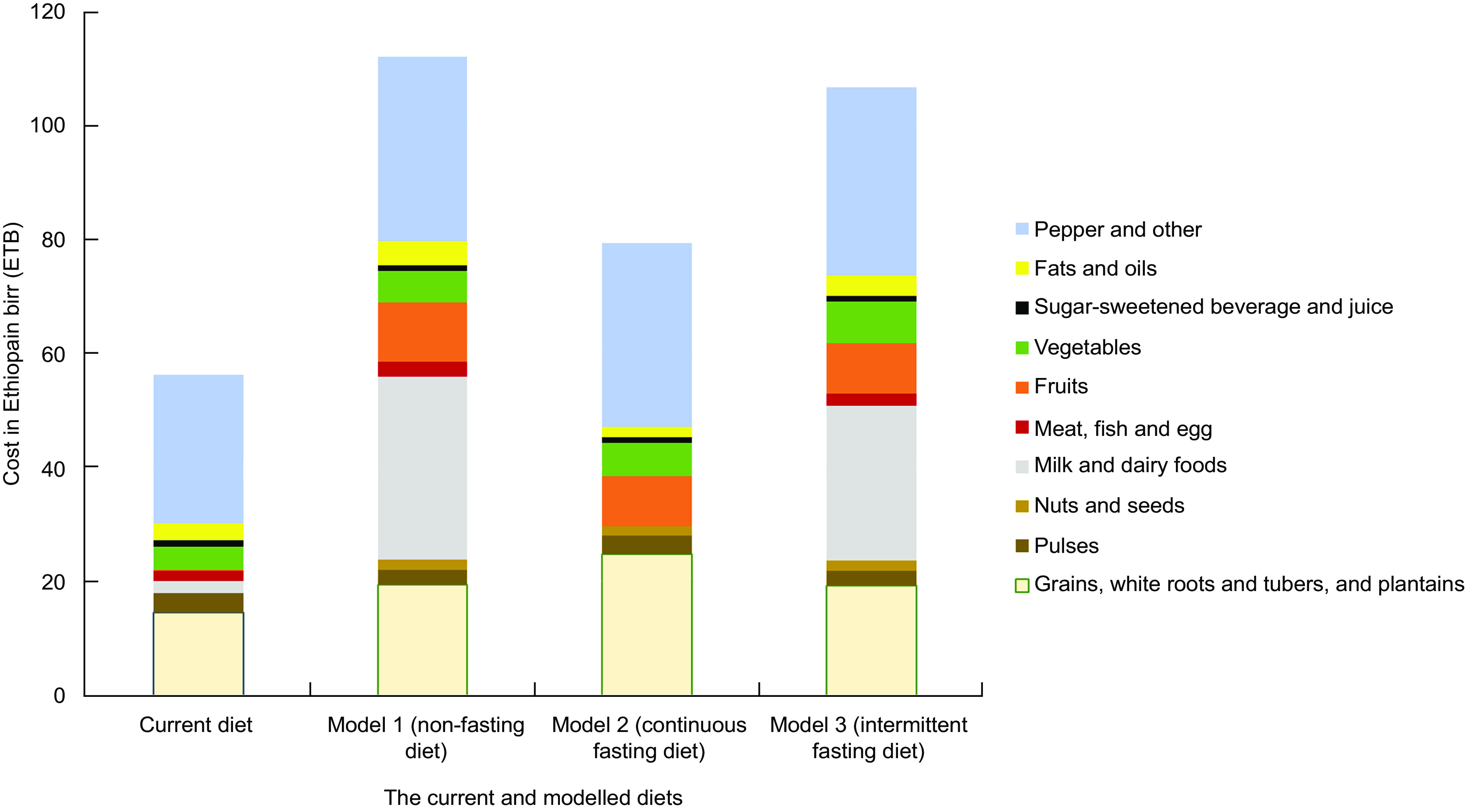
Average cost per food group per d for current and modelled diets. The Y-axis shows the cost of food group in birr per d

Models 1, 2 and 3 were calculated to cost 112 ETB (Ethiopian birr), 79 ETB and 105 ETB, respectively, whereas the current diet costed 56 ETB (Fig. 3). Ethiopia’s high cost of milk and dairy foods contributes to the cost of diet in the non-fasting and intermittent fasting diets. Fruits and vegetables make up a larger proportion of the cost in the modelled diets than the current diet. In the current and modelled diets, the ‘beverages, spice and other’ food group, which includes pepper, coffee, tea, other (non) alcoholic beverages, spices and salt, contributed to a larger proportion of the cost. Similarly, in all diets, the food group including cereals, roots and tubers contributed about 20 % of the cost. The overall cost of a continuous fasting diet (Model 2) is lower than that of the other two modelled diets but still higher than that of the current diet. The maximum daily cost of the proposed modelled diet is less than 120 ETB, or less than 3·5 USD per the exchange rate during the market survey.

## Discussion

The ultimate goal of dietary recommendations is to inform the public about healthy eating so that consumers can achieve their optimal energy and nutrient intake while minimising health risks^(31)^. In addition, the dietary recommendations will inform policies and programmes since the food supply and environments need to be supportive for dietary guidelines to be useful^(32)^. To support these recommendations, we aimed to optimise the current diet of Ethiopian women of reproductive age by targeting eleven micronutrients based on their public health importance in three different diet modelling scenarios (non-fasting diet, continuous fasting diet and intermittent fasting). The results showed that these three optimised diets meet daily energy and nutrient requirements, except for Ca and vitamin B_12_ in the continuous fasting model. The prevalence of women with inadequate intakes of the other targeted nutrients was less than 15 % in the modelled diets, which is a big improvement for Ethiopian women compared to the current diet. The current energy intake was close to the estimated daily energy requirement of Ethiopian adult women, that is, 2500 kcal/d^(33)^.

The diet for continuous fasting (Model 2) failed to meet the recommended intake of vitamin B_12_ and Ca as those two nutrients are mainly supplied by animal-source foods^(34,35)^. Vitamin B_12_ deficiency contributes to hyperhomocysteinemia, which has been identified as an atherothrombotic risk factor and a risk factor for dementia^(36)^. Vitamin B_12_ is also important for synthesising erythrocytes, which is important to transport oxygen to function in the nervous system^(37)^. A study indicated that fasting communities should have their vitamin B_12_ levels checked at least once a year to allow for timely intervention to the high risk of deficiency^(38)^. Annual monitoring of vitamin B_12_ levels might be feasible if it is integrated into medical care and focused on the most vulnerable populations and areas. However, maintaining an uninterrupted laboratory supply chain is a significant challenge in low-income countries like Ethiopia. Ca deficiency contributes to rickets in young children and osteoporosis in the elderly, especially in women^(39)^. Adequate intakes of Ca have also been shown to reduce the risk of pre-eclampsia and its associated complications, which are leading causes of maternal morbidity in pregnant women and preterm birth^(40)^. Although some plants contain easily absorbed Ca, the amount of vegetables required to meet the Ca requirements makes a plant-based diet unattainable for most people. Dietary components that reduce Ca absorption, such as salt, protein, phytate and caffeine, may be abundant in a continuous fasting diet^(41)^. A more comprehensive impact evaluation of vitamin B_12_ and Ca among the continuous fasting community in regular micronutrient surveys targeting other micronutrients at the population level can be helpful. Individuals who engage in fasting may benefit from vitamin B_12_ and Ca-fortified plant-based foods or supplements^(42)^. The fortification programme is currently in the initial planning stage, focusing on target micronutrients including Ca. However, there is a need to increase awareness about fortification and identify the appropriate vehicle of vitamin B_12_ fortification. Ca supplementation is only available for adolescent girls in specific areas.

Model 2, or the continuous fasting diet, was restricted to include only plant-based oils and fats, considered healthier than animal-derived oils and fats (except solid plant oils like palm oil)^(43)^. Additionally, it had twenty times (20 g *v*. 1 g) the amount of nuts and seeds compared to the current diet. Besides contributing to daily energy and nutrient requirements, a review revealed a lowered or no risk of NCD, such as CVD and type 2 diabetes, in subjects with nuts and seeds consumption up to 28 g/d^(44)^, an intake level equivalent to that in the modelled diets. Nuts and seeds are an integral part of the local Ethiopian diet, providing a good source of protein and plant-based fats that are essential during prolonged fasting periods. Although current production may not be sufficient, there is high production potential due to the favourable climate for nuts and seeds, which are one of Ethiopia’s main exporting commodities. The recommendation to promote nuts and seeds consumption was made in light of their significant potential in Ethiopia. Furthermore, the FBDG aim to inspire consumers to adopt healthier dietary patterns, with the promotion of nuts and seeds, dairy products, and fruit consumption as inspiration for healthier dietary choices. To make this recommendation feasible, government and all actors involved in the food systems need to take action.

The modelled intake of sugar-sweetened beverages, fats and oils did not differ from the current diet in our study. Low or moderate consumption of sugar-sweetened beverages, fats and oils (especially saturated fats) has been shown to reduce the risk of CVD and diabetes^(45)^. Moreover, in optimisation study finding, we compared the optimised quantity with the WHO’s recommendation that sugar-sweetened beverages intake should be limited to less than 10 % of total energy intake, which is roughly the same as the recommended amount from our study for a 2300 kcal/d diet. Based on this, the current and modelled diets can be considered healthy. The non-fasting diet (Model 1) showed large deviations from the current diet, with ten times the amount of milk and dairy foods (equivalent to one cup of milk per d) and thirty times the amount of fruit compared to the current diet. Despite these major dietary deviations, the resulting recommendations are consistent with Ethiopia’s current nutrition policies, which aim to fill those dietary gaps.

In the intermittent fasting situation (Model 3), nuts and seeds, milk and dairy products, and fruits are recommended to be consumed twenty-one times (21 g *v*. 1 g), nine times (298 g *v*. 31 g) and twenty-seven times (194 g *v*. 7 g) higher amounts than in the current diet, respectively. Consumption of those three food groups is also critical for meeting nutrient requirements and reducing the risk of diet-related NCD^(34)^. This is the first paper we are aware of that has attempted, for the intermittent fasting scenario, to compensate for inadequate Ca and vitamin B_12_ intake during the two fasting days by increasing the intake on the five non-fasting days per week. The assumption is that these nutrients can be stored in the body and used later, as indicated in the methods section. Human bodies contain about 1·2 kg of Ca, mainly stored in our bones and teeth. Plasma concentration is closely regulated, and the skeleton serves as a reserve store^(46)^. About 3–5 mg of vitamin B_12_ is stored in human bodies, with the liver accounting for 80 % of the total^(47)^.

The recommended daily intake of cereals in the modelled diets was equivalent to or slightly lower than the current consumption (608 g/d), which may be easier to adhere to as it does not require much emphasis on nutrition education and agricultural production when compared to other food groups such as fruit, dairy foods and nuts. Pulses and vegetables are also easy to incorporate because the amount in the modelled diets was comparable to the current diet. Most vegetable consumption in the current diet consists of onions and tomatoes, but a range of vegetables should be promoted since the consumption of a variety of green leafy and other types of vegetables is required to meet nutritional needs and will prevent NCD such as type 2 diabetes^(48)^. Nuts and seeds, milk and dairy products, and fruits require more emphasis on nutrition promotion and agriculture production so that the required increase in intake of these food groups can be achieved. The intake of fats and oils, sugar-sweetened beverages, salt, and alcohol should be limited while promoting a healthy diet.

The modelled diets were developed using 24-h quantitative intake data from 2-d recalls, which is a strength because the 2-d consumption provides a better approximation of the habitual intake than a single 24-HDR. The days of the week were evenly distributed across the entire study group, including working and weekend days and fasting and non-fasting days. The use of linear goal programming allows for optimising the intake of an individual and estimating the prevalence of inadequate nutrient intake at the population level. The method enabled us to include some of the current diet’s practical issues, such as the fact that some food groups are barely consumed in the current diet, for instance, dairy products and meat. Because of this, we assumed a specific proportion (75–100 %) of the GBD recommendation for the food groups consumed in minimal quantities and analysed the contribution to the optimal diet. The presented findings in Models 1–3 are based on 75–100 % of GBD recommendations for barely consumed food groups, since this gave us the maximum possible number of feasible solutions for diet optimisation.

The 24HDR and price data were only collected during the harvest season. Some studies indicate that seasonality influences the food availability in rural communities of Ethiopia, particularly for fruits and vegetables. This is a limitation of this study, as we do not consider seasonal variation in dietary intake or price volatility. According to a study published in 2020, the national average cost of foods to achieve nutrient adequacy during the observation period (2002–2016) was $1·34^(49)^. Over 1 year, the price peaked in late August, 2 months before the harvest season in November. The magnitude and timing of these price peaks reflect the limited degree to which different foods can substitute for one another to provide all the nutrients required throughout the year^(49)^. Another limitation relates to the generalisability of the study. The data collection did not cover the entire country and only focused on Ethiopia’s five major regions, so it did not include the pastoralist communities in other parts of the country. Since this dietary intake dataset is the most recent data presenting the current usual intake, it is possible to combine the findings from this study with other similar modelling work using previous dietary datasets to reach the final recommended amount for the Ethiopian FBDG.

The findings from this study are used as input for dietary recommendations at the population level. As a result, most of the model’s assumptions are at the population level, such as using EAR, 10–15 % deviation at the food group level and using GBD recommendations as a basis for modelling rarely consumed food groups.

Even though the modelled diets had a maximum cost of less than 120 Ethiopian birr (USD 3·5) per d in Ethiopia, at least half of the population would not be able to afford them due to poverty (https://dataafrica.io/profile/ethiopia/PovertyByGender). Using ten different definitions of a healthy diet published by the UN, the Member States, the range of the cost of healthy diets globally is between USD 3·27 and USD 4·57 per d, with a point estimate based on median costs of USD 3·75^(7)^. Intermittent and continuous fasting diets are less expensive than the EAT-Lancet cost of a global median of USD 2·84 per d in 2011, but the maximum cost of the non-fasting diet was higher^(50)^. The cost of the Model 1 and 3 diets was twice that of the current diet (56·4 ETB or 1·5 USD), and the cost of the Model 2 diet was 1·5 times that of the current diet. Note that the non-fasting diet has stricter requirements on vitamin B_12_ and Ca than the fasting diets. As a consequence, the continuous fasting diet has flexibility for lowering its cost at the expense of its vitamin B_12_ and Ca contents, if the prices and nutrient values of the remaining food items allow. This flexibility is less for the intermittent fasting diet, because it has to satisfy the vitamin B_12_ and Ca constraints at a weekly basis. According to a study on the cost of healthy diets, women’s earnings were consistently lower than men’s, making the diet relatively more costly^(7)^. As a result, the country’s ongoing socio-economic development, social SafetyNet programmes, food and nutrition policy and strategy, and the national food system transformation plan should all be strengthened to reduce the cost of diet and improve income^(42)^. The recommended healthy diets in this study will be included in the country’s FBDG, used as nutrition education, and a target-setting tool by various nutrition stakeholders. The model diet will need further translation into local menu development and to be experimented in practice.

### Conclusion

The model’s recommended diets (Model 1 or non-fasting, Model 2 or continuous fasting, and Model 3 or intermittent fasting) may be feasible for women of reproductive age because they are close to their current diets and fulfil their energy and nutrient demands. However, during continuous fasting, the proposed diet failed to provide enough Ca and vitamin B_12_, whereas the intermittent fasting diet compensated for those two nutrients on non-fasting days of the week. The recommended diet price is a point of attention. But considering the economic growth potential, current food and nutrition policy, and efforts to fill the gaps in providing affordable and healthy diets, the proposed diet might improve daily eating habits. Considering the quantity difference between the current and modelled diet, the implementation of the dietary guidelines in Ethiopia should aim to address the current food system challenges of the country, with special emphasis given to the consumption, availability, accessibility, and affordability of dairy foods, nuts and seeds, and fruits.

## Supplementary material

For supplementary material accompanying this paper visit https://doi.org/10.1017/S1368980023001374
